# Effect of Dog Presence on Stress Levels in Students under Psychological Strain: A Pilot Study

**DOI:** 10.3390/ijerph17072286

**Published:** 2020-03-28

**Authors:** Kristýna Machová, Radka Procházková, Mariana Vadroňová, Michaela Součková, Eliška Prouzová

**Affiliations:** 1 Department of Ethology and Companion Animal Science, Faculty of Agrobiology, Food and Natural Resources Czech University of Life Sciences, 16500 Prague, Czech Republic; maryvadronova@seznam.cz (M.V.); mis.souckova@seznam.cz (M.S.); elvinek@seznam.cz (E.P.); 2 Department of Statistics, Faculty of Economics and Management, Czech University of Life Sciences, 16500 Prague, Czech Republic; prochazkova@pef.czu.cz

**Keywords:** animal assisted activity, students’ health, stress, dog

## Abstract

As university students face many stressful situations, especially during the examination period, this study focused on the use of animal-assisted activities (AAAs) with a dog as a means of relieving students’ stress before a final exam. The aim was to determine whether a 10-min interaction with a dog affected subjectively evaluated stress and mood, objective blood pressure, and heart rate. Ninety-three female students (mean age = 22.5 years; standard deviation = 3.8 years) were divided into three groups according to their preference. The first group underwent AAAs (*n* = 26), the second group chose a relaxation technique (*n* = 28), and the last one was a control group (*n* = 39). Physiological values were measured using a pressure gauge and the subjective feelings of stress and mood were evaluated by the Likert scale 1–5. The AAA group showed significant improvement after 10 min of interaction in both mood and stress, with no change in heart rate and blood pressure. The remaining groups showed a significant decrease in blood pressure, but not in heart rate, with different evaluations of mood and stress. AAAs with a dog appear to be effective in improving students’ mood and stress without affecting their physiological parameters.

## 1. Introduction

Stress is defined as a certain response of an organism to tension, as well as the excitement of the mind and body [1]. The consequences of stress are one of the main causes of death along with heart disease, cancer, liver and lung disease, or suicide [2]. The stress response is a major factor in the development of many complications, including cardiovascular and mental illnesses [3]. During the stress response, stress hormones are released, and they cause an increase in blood pressure and heart rate [4,5]. These parameters may vary during the day and this variation is impacted by many factors [6].

The nature of the biological stress response is considerably affected by interindividual differences in stress perception, processing, evaluation, and management. Lower doses of stress can be motivational and encourage people to perform better. This type of stress is called eustress—“good stress”. Hormones released in response to this stress can improve memory, cause higher concentration, and affect the biological processes involved in enhancing immunity [7]. An acute or short-term stress response induces a rapid transfer of immune cells to different parts of the body. This stress-induced transfer of leukocytes to the target organs will increase the speed, efficiency, and regulation of immune reactions [8]. Mental tenacity increases and the organism becomes stronger than before the stressful experience [9].

Long-term and unmanageable stress (distress), however, affects the body negatively compared to eustress. Along with harmful effects on mental and physical health, the consequences of long-term stress include an increased risk of premature death [10]. The aforementioned excessive stress is associated with chronic anxiety, psychosomatic diseases, and numerous additional emotional difficulties [11]. The potential outcomes could be burnout syndrome, clinical hypertension, and ischemic heart disease [12].

For students, the effects of moderate stressors can have a positive impact, motivate and inspire them, as well as enhance their creativity. However, higher stress levels can not only be demotivating, but may lead to sleep disorders, depression, problematic substance use, and suicide [13,14]. An increasing number of undergraduates face depression, anxiety, and other mental health problems as a result of various factors such as family relationship problems, drug and alcohol experimentation, pressure from high-performance expectations, and stress associated with examination periods. According to the estimates, 85% of university students (usually 18–25 years old) experience high levels of stress, over 50% suffer from depression, and up to 11% have suicidal ideation [15]. One of the ways to help students cope with stress could be animal-assisted activities (AAAs), which currently take place in some social institutions, schools, and hospitals [16,17]. These activities can be performed with the participation of various animals, such as small pets, livestock, horses, and llamas [18,19].

AAAs may bring numerous positive effects, such as improving mood and health [20], reducing stress [21] and depression [22], increasing the incidence of achievements of rehabilitation goals [23], and increasing social interactions [24]. AAAs can also be used in the treatment of severe mental illnesses, such as post-traumatic stress disorder [25]. This activity is increasingly more often taking place in pre-schools and schools [26]. The purpose of AAAs in schools is to encourage children to increase their social interactions, participate in education, and modify their behavior. An equally important aim of AAAs is also its effect as a preventive factor against risky behavior, and, at the same time, a mediator facilitating introverted students to interact with classmates and get more involved in the course of education [27]. The presence of a specially trained dog may have a beneficial effect on the climate of the whole class [28].

For university students, it is possible to use AAAs directly within the campus programs as group or individual activities to reduce stress and increase happiness and energy levels [29]. It has been observed that even one AAA with a dog can bring much greater benefits to students than looking at pictures of dogs [30] or watching a video of a dog [31]. The reason for increased stress in students can be not only the difficulty of the studies, but also separation from their family and familiar environment. AAAs can then have an impact on reducing homesickness while living on campus and may increase students’ well-being and satisfaction in life [32].

For students at all levels of education, examination periods are regular sources of stress, which are further associated with increased anxiety and depression, elevated cortisol levels, and immune dysregulation. Moreover, stress caused by examinations is associated with poorer academic performance [33,34,35]. This study aimed to evaluate the influence of the dog’s presence on students in a stressful situation by using physiological parameters and subjective evaluation of stress and mood. Our hypothesis was that after interacting with a dog, students would feel less stressed, their mood would improve, and their blood pressure and heart rate would be lower than if they participated in relaxation activities or did not participate in any extra activity.

## 2. Materials and Methods

### 2.1. Participants

Ninety-three students were recruited for this study. The participants in the experimental groups were aged between 19 and 44 years (mean = 22.5 years, median = 22 years, modus = 21 years, standard deviation of age = 3.8 years, age coefficient of variation = 17.01%). Only women were included in the study, as the presence of male students in the given year was rather rare at this institution. Our sample of female students was recruited through the given year’s Facebook page, where they were informed about the possibility of participation in the research. Handlers with dogs and the people providing other options of relaxation participated in 16 announced exam dates. Each day, the students were once again approached directly with the opportunity to participate in this study. At that time, the students chose the tested alternative that they considered most beneficial for them. All of the participants were awaiting an exam in genetics, which is generally considered to be one of the most difficult exams at this institution. The exam took place in the second year of bachelor’s degree studies and all of the students attempted to pass the exam for the first time. The criteria under which the students were excluded from the study were no reported stress over exams and/or not being fond of animals. Animal ownership was not an exclusion criterion.

None of the students reported mental problems, health problems with blood pressure or heart rate, or regular use of medication that could affect the monitored physiological values. No other specific illnesses were taken into account as a negative factor for the overall study. The conditions of the research corresponded to real practice.

The project was approved by the Ethics Committee of the Czech University of Life Sciences in Prague. The study and its methodological procedure adhered with the requirements of the European Union and Czech legislation (Act No. 246/1992 Coll. on animal protection as amended by Act No. 162/1993 Coll.).

### 2.2. General Procedures and Measures

The participants were divided into three groups based on their preferences. Group A (26 students) participants individually underwent an AAA, which was attended by three trained dogs, each with their handlers. The dogs that performed interactions with students had passed animal-assisted interaction (AAI) examinations, which test a dog’s behavior and its suitability to perform this activity. The dogs were selected on the basis of long-term cooperation with the university in this field. All handlers had the same instructions, namely, to focus interactions with the dogs on calming and relaxing the students. AAAs were realized in a quiet area in the proximity of the office where the exam was held. The sessions were individual and took place on blankets on the ground in order to be close and in possible physical contact with the dog. The handlers were instructed to familiarize the student with the dog. The students could sit comfortably in their most suitable position and pet the dog. If the students themselves actively started a conversation with the handler, then the handler calmly talked with the students about the animal or animals the students had at home. Absolute silence was not specifically chosen in this case as it might be unpleasant for some students. The final form of AAA therefore depended on the particular student and their needs. The dog was calm at all times, either sitting or lying with the student. The aim of AAAs was to create a pleasant and friendly environment with the main focus on the animal and to alleviate the stress from the upcoming exam in the student–dog–handler triad. Other students were able to observe AAAs but could not interfere. Group B (28 students) had the opportunity to unwind and relax using aids such as kinetic sand, anti-stress cubes, anti-stress coloring books, and a phone with music. Group C (39 students) was not affected.

Each student completed a record sheet, in which they familiarized themselves with the study and signed informed consent. The sheet included demographic questions about gender, age, height, weight, and possible allergies. The next section of the sheet focused on the subjective evaluation of mood, i.e., the student’s emotional state of mind during this experience, and subjective level of stress. These two emotions were scored on a five-point Likert scale with the instruction that the lowest score indicates the best mood and least amount of stress, and the highest score (5 points) indicates the worst mood and highest amount of stress. This assessment was done by the student before and after the interaction without the presence of a dog.

Every student was measured and asked about mood and stress before and after 10 min of the given activity or, in the case of the control group, no activity. Blood pressure and heart rate were measured with the pressure gauge Omron M3. The measurements were done three times before and three times after the interaction, and arithmetic mean was calculated from these values. The total measurement of one student took around 15–20 min (about 1 min of pulse and blood pressure measurement (3x), followed by a 10-min interaction, and then about 1 min of measurement (3x) + time spent completing the questionnaire). All measured values were recorded on the record sheet and then statistically evaluated. We carried out all the measurements at the same time each day to minimize possible variations because the tested parameters can be influenced by many factors, including the time of the day.

### 2.3. Dogs Involved in the Study

In total, three therapy dogs of different breeds were present: a Bolognese dog, a border collie, and a Rhodesian ridgeback. All dogs were certified to perform AAAs, and their handlers practiced AAAs as long-term volunteers and even on a professional level. The test that these dogs underwent included an evaluation of their behavior toward humans and other animals. It also included an assessment of the dogs’ behavior in an environment where an AAA was performed, as well as their obedience. An integral part of these tests was the evaluation of the handler and their approach to the users, relationship with the dog, and knowledge of AAAs, ethology, and welfare of dogs. All teams of dog and handler passed this test with an excellent rating. The dogs showed friendly behavior toward people and were relaxed while in contact with the students. Throughout the AAA time, the dogs had access to water and the opportunity to leave the interaction if they needed to. At any sign of discomfort, the handlers were instructed to end the interaction immediately. All animals included in the study were under veterinary supervision and were dewormed and vaccinated in general accord with the schedules recommended in the Czech Republic.

### 2.4. Data Analysis

The measured data were analyzed by groups (A, B, and C) depending on the way students tried to relieve their stress before the difficult test: group A used animal-assisted activities with a dog (AAA), group B had a choice of relaxation elements, and group C was not affected.

The STATISTICA 13.2 software (StatSoft, Tulsa, OK, USA, version Cz. 7) for Windows was used for statistical analyses. At baseline, an exploratory data analysis was conducted to verify the assumptions for subsequent processing (such as sampling independence, homogeneity, and distribution normality). The premise was the normal Gaussian distribution, which was assessed by the Shapiro–Wilk test and further evaluated on histograms and normalized probability diagrams.

Analyzed data on students’ heart rate and blood pressure showed normal Gaussian distribution. Therefore, a parametric paired t-test was used to test and generalize the conclusiveness of the measured values before and after the paired data (pretest–post-test research) and the dependence of the sample sets. The non-parametric Wilcoxon test for two dependent samples was used to assess and test the statistical significance of changes in subjective stress and mood status measured using the Likert scale. The reason was the discontinuity of the examined statistical sign and its ordinal character.

Repeated measures ANOVA was used to test and generalize the significance of the differences in the measured values between all three groups of students (A, B, and C) after verifying the assumptions (distribution normality—Shapiro–Wilk test, homoscedasticity—Levene dispersion homogeneity test). The Scheffe test was used for subsequent post-hoc analysis.

The magnitude of changes in measured values to baseline measures was evaluated and tested by using a simple linear regression and correlation analysis. Tests of significance of regression models were performed, and the strength of dependence was measured and assessed by correlation coefficient (r) and coefficient of determination (r^2^).

Statistical significance was set at *p* < 0.05. Box and frequency graphs were used to visualize the results of statistical analyses and tests.

## 3. Results

**Figure 1:**
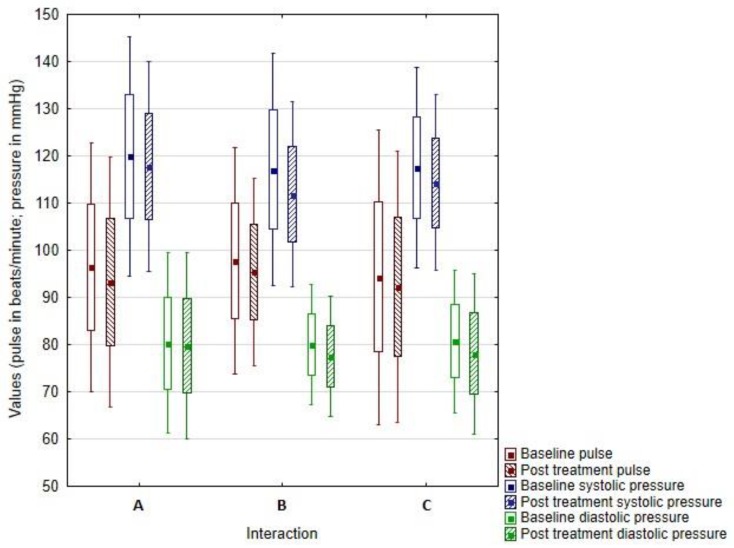
Changes in heart rate and blood pressure in groups **A** (animal-assisted activities with a dog), **B** (selected relaxation technique), and **C** (no influence).

No significant reduction in systolic (*p* = 0.362) or diastolic pressure (*p* = 0.695) was observed in group A, i.e., in the presence of a dog. The heart rate reduction was close to the level of significance, but a statistically significant difference was not shown (*p* = 0.059). The results of the measurements are summarized in Figure 1, which show changes in heart rate and blood pressure in groups A, B, and C. In the evaluation of mood before and after the intervention, a significant improvement (*p* < 0.001) was observed. On average, respondents reported a one-point mood improvement (min difference = 0, max difference = 3). None of the respondents reported a deterioration in mood. The subjective evaluation of stress also showed a significant decrease (*p* = 0.002), with the average difference (decrease in the feeling of stress on the 1–5 point scale) being 0.731 points (min difference = −1, max difference = 2).

In group B, i.e., in the group using relaxation techniques, a significant decrease in systolic (*p* = 0.016) and diastolic pressure (*p* = 0.015) was observed. No significant decrease in heart rate was observed (*p* = 0.108). This group also showed cut-off values of mood change (*p* = 0.059) and decrease in stress (*p* = 0.051), but no statistically significant difference was confirmed. On average, respondents from this group reported a 0.214-point mood improvement, with the vast majority (71%) showing a zero-mood change (modus = 0 and frequency = 20, min difference = −1, max difference = 1). Similar results were reported for stress. The mean difference (decrease in the feeling of stress on a scale of 1–5) was 0.286 points (min difference = −1, max difference = 2), and most respondents (61%) showed no change in the subjective evaluation of the feeling of stress.

**Figure 2:**
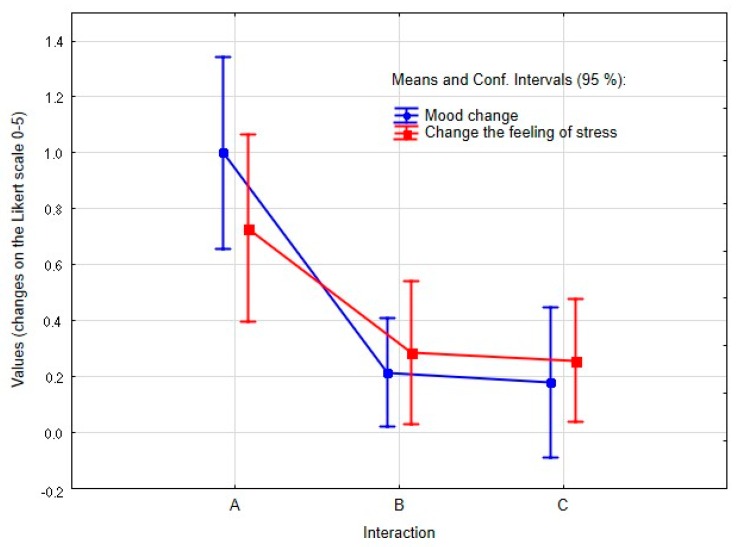
Changes in subjective evaluation of mood and stress in groups A (animal-assisted activities with a dog), B (selected relaxation technique), and C (no influence).

In group C, the non-influenced group, a significant decrease in diastolic pressure (*p* = 0.007) and systolic pressure (*p* = 0.010) was observed. The reduction in heart rate was at the significance threshold (*p* = 0.058). No significant mood improvement was found in this group (*p* = 0.201). On average, respondents from this group reported a 0.18-point mood improvement, with 62% showing a zero-mood change (difference mode = 0 and frequency = 24, min difference = −2, max difference = 2). In contrast, statistically significant stress relief was demonstrated (*p* = 0.044). The mean difference (decrease in the feeling of stress on a scale of 1–5) was 0.256 points (min difference = −1, max difference = 2). Figure 2 summarizes the results of subjective evaluation of mood and feeling of stress in all three groups.

**Figure 3:**
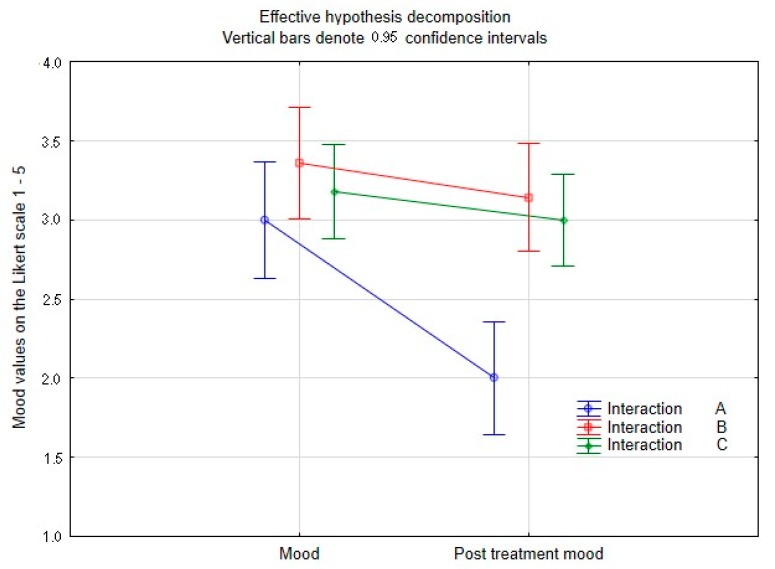
Summary of subjective mood evaluation in groups A (animal-assisted activities with a dog, B (selected relaxation technique), and C (no influence) after performed activities.

**Figure 4:**
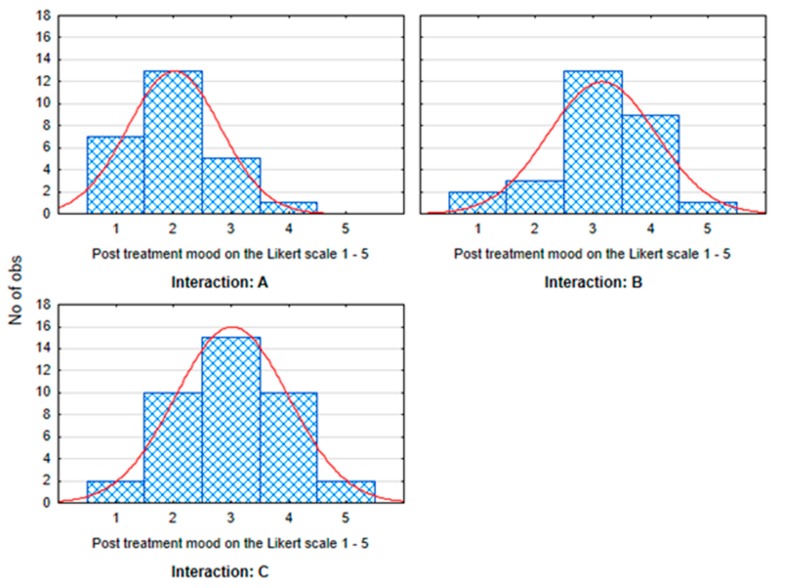
Categorized histograms showing the distribution of subjective mood values in groups A (animal-assisted activities with dog), B (selected relaxation technique), and C (no influence) after performed activities. 1, best mood; 5, worst mood.

The multivariate tests (Table 1), where the significance of the differences between all three groups of students (A, B, and C) was assessed simultaneously, showed that there was no statistically significant difference between heart rate, systolic pressure, diastolic pressure, and stress assessment. There was a significant difference in mood (*p* = 0.004). In this variable, group A differed significantly from the other groups (B and C) (A versus B, *p* = 0.007; A versus C, *p* = 0.027). There was no statistically significant difference between B and C (*p* = 0.750). The previously mentioned results suggested that the influence of interactions (and the method of relaxation) can be described as statistically significant only for subjective mood evaluation (Figure 3). For detailed distribution of measured mood values in individual evaluated groups, see Figure 4.

The results of repeated measures ANOVA further revealed that all the measured variables, regardless of the method of relaxation (A, B, C) used, showed a significant change (decrease) of measured values (heart rate, *p* = 0.002; systolic pressure, *p* < 0.001; diastolic pressure, *p* = 0.004; mood, *p* < 0.001; stress, *p* < 0.001).

We also tested if the baseline measure predicted the change scores, and if they could be a bigger effect in students who were stressed by the exam compared to students who were not much affected. In most cases (Table 2), statistically significant, direct, weak to moderate dependence between the baseline level of the measured value and the subsequent magnitude of the change (decrease of the measured value) was observed.

Thus, for mood and stress parameters where the difference between baseline and post-treatment was most pronounced, the correlation between initial stress and the resulting shift was moderate. In the case of group A, it can be stated, based on the value of the determination coefficient (r^2^), that the positive mood changes were influenced by the mood before the interaction by 33.6%. In the feeling of stress, positive changes (reduced feeling of stress) were influenced by the initial stress level by almost 21%.

## 4. Discussion

Higher education institutions are aware of difficulties that may burden students and seek ways to help students to find appropriate means to relieve stress and develop effective coping strategies. One possible means seems to be animal-assisted activities (AAAs), which provide students with the opportunity to interact with dogs [36]. In our study, we focused on assessing stress using physiological parameters of heart rate and blood pressure. At the same time, mood and stress were subjectively evaluated using the Likert scale, similar to the study of Pendry et al. 2018 [37].

In our study, the group with the dog did not show a significant reduction in systolic or diastolic pressure. Delgado et al. [38] reported a decrease in the physiological values of the students, but not in the value of diastolic pressure. In their study, the most significantly reduced physiological value was salivary cortisol, which was not measured in our study. On the contrary, the study by Jarolmen and Patel [39] noted that after performing AAAs with college students before and after the final exam, there was a significant difference in both systolic and diastolic pressure.

Our results are fully consistent with Delgado et al. [38] in regard to mood evaluation and subjective stress evaluation. In both studies, a statistically significant improvement in mood and a reduction in stress perception were observed, provided the students had the opportunity to interact with a dog. In our study, stress reduction was observed in the evaluation of baseline and post-treatment values. However, no difference was detected when compared with the other tested groups. In the case of mood, there was a significant difference in the evaluation of baseline and post-treatment values as well as in comparison with the post-treatment values of the other tested groups. According to Barker et al. [40], the events on campus with dogs represent a financially and readily available activity to reduce perceived, but not physiological, stress for college students before the final examinations. In the Jarolmen and Patel study [39], all the participants reported that they had enjoyed the time spent interacting with dogs, with some feeling more relaxed and less anxious.

The Polheber and Matchock study [41] compared groups with social support in the form of a dog or a friend, and a control group without support. It was reported that social support in the form of an unknown dog reduced salivary cortisol levels compared to the support of a friend and the control group. In a way, similar results were observed in our study, where mood and subjectively assessed stress improved in the group of the students who interacted with a dog, but not in the group with access to various relaxation techniques; and in the group with no activity, only subjectively assessed stress improved. Furthermore, in our study, only blood pressure (both systolic and diastolic) was reduced in the relaxation technique group (B). However, it is possible that with a higher number of students, heart rate would be statistically significantly reduced due to the close approximation of the significance level. A possible explanation is that our students lacked their preferred relaxation method.

In the control group (C) that was not affected, a statistically significant decrease in diastolic pressure, but not systolic pressure and pulse, was observed. There was also an improvement in stress, but no significant decrease in mood was observed. The authors do not know the reason for the mood change and have no clear explanation for it.

In our study’s experimental group, the interaction with the dog lasted 10 min, as compared to, for example, the study conducted by Wood et al. [42], where groups of six students interacted with two dogs for 15 min. The short interaction time in our study was based on practical applicability, where it was necessary to think about the welfare of therapeutic animals as well as the number of possible interventions provided. Wood et al. [42] observed that anxiety improved after the AAA, as it did in our study. Compared to their study, however, we did not see an improvement in blood pressure (BP), which they did. That is probably because in the Wood et al. [42] study, 24% of the students had clinical hypertension (systolic BP over 140 mmHg) in the pre-intervention time period. At the post-intervention time point, 20% of students had clinical hypertension (systolic BP over 140 mmHg). In a recent study by Binfet et al. [36], AAAs significantly reduced self-reported stress. A total of 1960 participating students had the opportunity to choose the length of time spent with a dog. The average preferred time to reduce stress via AAAs was 35 min. Grajfoner et al. [43] concluded that even a short 20-min session with a therapeutic dog could be an effective alternative intervention to improve students’ well-being, anxiety, and mood. Determining the optimal duration of therapy, therefore, appears to be an appropriate topic for future studies to ensure maximum efficacy of therapies.

An interesting finding reported by Grajfoner et al. [43] was that the presence of the handler together with the dog seemed to have a neutral to a somewhat negative effect on the mood of the participants. Much more positive mood improvement was observed in interactions where the participants interacted only with the dog than in the interactions where both the dog and the handler were present. The possibility to interact only with the dog was not provided in our study. However, there was a rotation of three handlers who had different breeds of dogs. Therefore, the authors do not rule out that the experience of the students could be influenced by the handler or by the dog breed.

Many studies point to the fact that the presence of a dog affects stress reduction [28]. Similarly, the effect of the presence of a dog on the reduction of heart rate and blood pressure has been observed [44,45,46]. The results of these studies clearly point to the fact that ownership or even the mere presence of a dog in the proximity of a person improves mood and reduces stress. Although this study did not show physiological stress reduction, the students’ subjective feeling of stress reduction was noticeable. The results show that AAAs can have a positive effect on students’ mental state before exams. In our case, they were students who showed adequate stress to the situation. It would be interesting to evaluate the influence of AAAs before examinations in students who suffer from anxiety, have communication problems, or suffer from inadequate nervousness or demands on themselves. For these students, AAAs could be even more effective. This is a method that is easy to integrate into university programs and, while paying careful attention to avoid the risk of overloading therapeutic dogs, could bring much relief and joy to both students and their handlers and dogs.

### Limitations

The main limitation of this study was the environment in which the measurements took place, as they were not laboratory conditions. The measurements were carried out directly on the premises where students waited for the exam. Providing a separate room for the relaxation activities and the interaction with the dog to ensure a calm and more intimate atmosphere could produce different results. However, this study aimed to evaluate results from real practice and from the environment and situation where AAAs may take place. Another limitation could be that the students were assigned to groups that were not selected randomly. In this instance, the authors assumed that students would best evaluate what kind of distraction would be best for them. It should be noted that the use of caffeine and nicotine might have influenced the results. However, we could not instruct the students not to use these substances as they might serve as the students’ coping strategy. The presence of three different dogs with three different handlers could affect the results obtained. We are aware that the personality of the handler and the nature of the dog can affect the measurement. For this reason, we chose professional handlers with equally professional dogs who were given clear instructions to alleviate this potential bias as much as possible.

## 5. Conclusions

This study highlights the potential benefits of student interaction with therapeutic dogs at a university before the final examination. The premise of the study was to improve mood and reduce stress that was not only subjectively perceived, but also objectively measured by a pressure gauge. Compared to the control group and the group with other activities, brief interventions with a dog had a significant effect on subjective mood improvement. When comparing the baseline and post-treatment values in the three groups, a reduction in subjectively perceived stress and mood improvement was observed in the AAA group. No effects on objectively measured parameters such as heart rate and blood pressure were observed when interacting with a dog. In the remaining two groups, a decrease in blood pressure was observed, with all levels of blood pressure and heart rate being physiological at all times. Although it was a short 10-min interaction, the results clearly indicated a possible positive impact on university students. Therefore, we recommend considering this low-cost and readily available method to universities to relieve students in stressful periods; however, the students should be allowed to choose the most appropriate coping strategy for them.

## Figures and Tables

**Table 1:** Testing the significance of the difference between groups.

Observed Parameter	Intergroup Differences
Heart rate	*p* = 0.596
Blood pressure, systolic	*p* = 0.272
Blood pressure, diastolic	*p* = 0.836
Mood	*p* = 0.004 *; A versus B, *p* = 0.007 *; A versus C, *p* = 0.027 *; B versus C, *p* = 0.750
Stress	*p* = 0.254

* Statistically significant difference was observed between groups. A *p*-value < 0.05 is considered a statistically significant difference.

**Table 2:** Summary of correlation analysis results—baseline measured value (explanatory variable) and change of measured value score (dependent variable).

Interaction	Heart Rate	Blood Pressure Systolic	Blood Pressure Diastolic	Mood	Stress
**Group A** Animal-assisted activities with dog	*p* = 0.145; r = 0.294	*p* = 0.002 *; r = 0.581; r^2^ = 33.76%	*p* = 0.095; r = 0.334	*p* = 0.002 *; r = 0.580; r^2^ = 33.64%	*p* = 0.019 *; r = 0.458; r^2^ = 20.98%
**Group B** Selected relaxation technique	*p* = 0.002 *; r = 0.565; r^2^ = 31.93%	*p* < 0.001 *; r = 0.636; r^2^ = 40.45%	*p* = 0.034 *; r = 0.402; r^2^ = 16.16%	*p* = 0.025 *; r = 0.424; r^2^ = 17.98%	*p* = 0.010 *; r = 0.481; r^2^ = 23.14%
**Group C** No influence	*p* = 0.015 *; r = 0.387; r^2^ = 14.98%	*p* = 0.001 *; r = 0.506; r^2^ = 25.60%	*p* = 0.213; r = 0.204	*p* = 0.018 *; r = 0.376; r^2^ = 14.14%	*p* = 0.136; r = 0.243
All groups	*p* < 0.001 *; r = 0.400; r^2^ = 16%	*p* < 0.001 *; r = 0.557; r^2^ = 31.03%	*p* = 0.004 *; r = 0.295; r^2^ = 8.7%	*p* = 0.001 *; r = 0.333; r^2^ = 11.09%	*p* < 0.001 *; r = 0.340; r^2^ = 11.56%

* Statistically significant correlation was observed. A *p*-value < 0.05 is considered statistically significant.
